# Ge_2_Sb_2_Te_5_ p-Type Thin-Film Transistors on Flexible Plastic Foil

**DOI:** 10.3390/ma11091672

**Published:** 2018-09-09

**Authors:** Alwin Daus, Songyi Han, Stefan Knobelspies, Giuseppe Cantarella, Gerhard Tröster

**Affiliations:** Electronics Laboratory, Department of Information Technology and Electrical Engineering, 8092 Zürich, Switzerland; songyi.han@polytechnique.edu (S.H.); kstefan@ife.ee.ethz.ch (S.K.); gcantare@ife.ee.ethz.ch (G.C.); troester@ife.ee.ethz.ch (G.T.)

**Keywords:** GST, thin-film transistor, flexible electronics, P-type semiconductor, crystalline materials, amorphous materials, phase-change materials, germanium, antimony, tellurium

## Abstract

In this work, we show the performance improvement of p-type thin-film transistors (TFTs) with Ge${}_{2}$Sb${}_{2}$Te${}_{5}$ (GST) semiconductor layers on flexible polyimide substrates, achieved by downscaling of the GST thickness. Prior works on GST TFTs have typically shown poor current modulation capabilities with ON/OFF ratios ≤20 and non-saturating output characteristics. By reducing the GST thickness to 5 nm, we achieve ON/OFF ratios up to ≈300 and a channel pinch-off leading to drain current saturation. We compare the GST TFTs in their amorphous (as deposited) state and in their crystalline (annealed at 200 °C) state. The highest effective field-effect mobility of 6.7 cm${}^{2}$/Vs is achieved for 10-nm-thick crystalline GST TFTs, which have an ON/OFF ratio of ≈16. The highest effective field-effect mobility in amorphous GST TFTs is 0.04 cm${}^{2}$/Vs, which is obtained in devices with a GST thickness of 5 nm. The devices remain fully operational upon bending to a radius of 6 mm. Furthermore, we find that the TFTs with amorphous channels are more sensitive to bias stress than the ones with crystallized channels. These results show that GST semiconductors are compatible with flexible electronics technology, where high-performance p-type TFTs are strongly needed for the realization of hybrid complementary metal-oxide-semiconductor (CMOS) technology in conjunction with popular n-type oxide semiconductor materials.

## 1. Introduction

Flexible electronics have received increased attention in the last years, promising great innovations in emerging display technology [1], healthcare [2,3,4], human-machine interfaces [4,5], textiles [6] and flexible sensor systems for Internet-of-Things (IoT) applications [7]. Especially, the latter requires high-frequency operation (13.56 MHz) to enable wireless data transmission via radio frequency identification (RFID) and near-field communication (NFC). Oxide semiconductor based thin-film transistors (TFTs) offer large carrier mobilities (>10 cm${}^{2}$/Vs), which enable these high frequencies, and additionally provide low off-currents and large area uniformity [8,9,10,11]. Thus, recent reports have shown that unipolar circuit technology based on oxide semiconductors can be employed for NFC and RFID applications [12,13]. However, the use of complementary metal-oxide-semiconductor (CMOS) technology could dramatically improve the power consumption, gain, noise immunity and circuit design of these systems [14].

Recently, several groups of materials have been studied to realize flexible hybrid CMOS together with n-type oxide semiconductors involving p-type SnO [14] or carbon nanotubes [15,16,17,18]. So far only few works have considered GeSbTe (GST) compounds as channel materials for TFTs [19,20,21,22,23,24,25], despite their p-type semiconducting properties [26,27], potentially large hole mobilities between 10–100 cm${}^{2}$/Vs [27,28], as well as low-temperature processability. Notably, the material has been extensively studied for phase-change memory applications [29] and is commercially applied in rewritable optical discs [30]. Prior works on GST TFTs have found difficulties in the ON/OFF current modulation and the output characteristics did not show saturating drain currents (I${}_{D}$) [19,20,21,22,23,24,25]. In this work, we study GST TFTs on a flexible substrate and demonstrate performance improvements through downscaling of the GST thickness to 5 nm. For this GST thickness, we find ON/OFF ratios up to ≈300, which represents a 15-fold improvement compared to prior work. The thickness reduction also leads to a pinch-off of the semiconductor channel, resulting in a saturating I${}_{D}$ in TFT output characteristics. Furthermore, we compare the device characteristics before and after thermal annealing above the crystallization temperature, which leads to an increased conductivity and mobility for GST thin-films ≥10 nm.

## 2. Experimental

### 2.1. Device Fabrication

The bottom gate TFTs were processed on a flexible 50 μm thick polyimide substrate. First, the substrate was cleaned in isopropanol and acetone by sonication, followed by a baking at 200 °C in an air oven for 24 h. Then, a SiN${}_{X}$ buffer layer was applied on both sides of the substrate by plasma-enhanced chemical vapor deposition at 150 °C. The Ti/Au/Ti (5/30/5 nm) gate electrode was deposited by electron-beam evaporation and structured by lift-off. The surface was cleaned by 1 min of ultraviolet (UV) ozone treatment and subsequently a 20-nm-thick Al${}_{2}$O${}_{3}$ gate dielectric was deposited by thermal atomic-layer deposition at 150 °C. The amorphous GST p-type semiconductor layer was direct-current (dc) magnetron sputtered from a Ge${}_{2}$Sb${}_{2}$Te${}_{5}$ target at room temperature (see reference [31]) and structured by lift-off. To avoid damage to the Al${}_{2}$O${}_{3}$, this lift-off was executed using Poly(methyl methacrylate) (PMMA) photoresist and deep-ultraviolet lithography. Afterwards, via holes were wet chemically etched into Al${}_{2}$O${}_{3}$. Finally, the Ge/Ni/Au (5/5/30 nm) source/drain electrodes were electron-beam evaporated and patterned by lift-off. The post-fabrication crystallization was performed in a vacuum oven at a pressure *p* = 200 mbar and 200 °C for 1 h.

### 2.2. Material Characterization

The *x*-ray diffraction (XRD) measurements of the GST thin-films were performed with a D2 Phaser 2nd Gen, Bruker, Billerica, MA, USA (30 kV, 10 mA, wavelength λ = 0.154 nm).

The Hall measurements were done on a Nanometrics HL5500 Hall System at ambient temperature at a magnetic field of 0.32 Tesla. The GST thin-films had Ge/Ni/Au (5/5/30 nm) contacts and were simultaneously fabricated with the TFTs. The current for 10 nm, 20 nm and 50-nm-thick crystallized GST films was set to 0.01 mA, 0.5 mA and 1 mA, respectively. The amorphous films were too resistive to perform Hall measurements.

### 2.3. Thin-Film Transistor Characterization

All electrical measurements were performed on a probe station (Rucker Kolls, Inc., Milpitas, CA, USA) at ambient conditions with a B1500A semiconductor device analyzer (Agilent Technologies, Santa Clara, CA, USA). Bias stress was applied on the TFTs with a constant gate-source voltage V${}_{\mathrm{GS}}$ = −5 V and a constant drain-source voltage V${}_{\mathrm{DS}}$ = −100 mV, immediately followed by a double-sweep measurement of the transfer characteristic.

## 3. Results and Discussion

**Figure 1:**
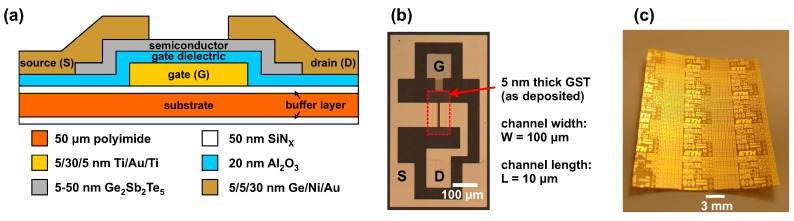
Thin-film transistors (TFTs) on flexible plastic substrates. (**a**) Schematic TFT cross-section. (**b**) Optical micrograph of a TFT top view. (**c**) Photograph of fully fabricated devices on 50 μm thick polyimide.

**Figure 2:**
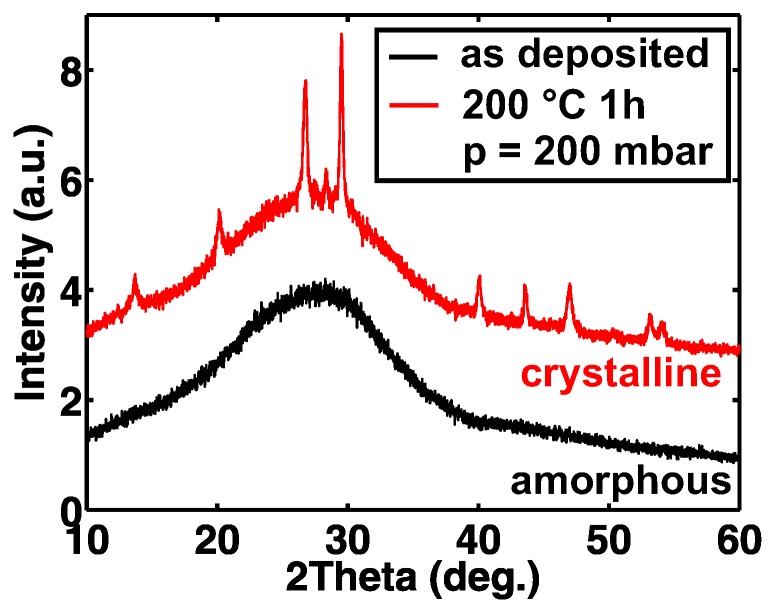
X-ray diffraction of 100-nm-thick GST on quartz glass.

The GST TFTs have been fabricated on a free-standing flexible 50 μm thick polyimide foil as described above. Here, different GST thicknesses between 5 and 50 nm are studied. A schematic cross-section of the TFTs is shown in Figure 1a. A micrograph of a fully fabricated TFT with 5-nm-thick GST (as deposited) is displayed in Figure 1b. Figure 1c shows a photograph of the flexible polyimide substrate after the device fabrication. The device performance is tested in the amorphous (as deposited) state and after annealing at 200 °C in a vacuum oven. To confirm the phase-change of GST upon annealing, we performed XRD measurements. Figure 2 reveals a mixed face-centered cubic (fcc) and hexagonal close-packed (hcp) crystal structure of GST after annealing [32,33].

**Figure 3:**
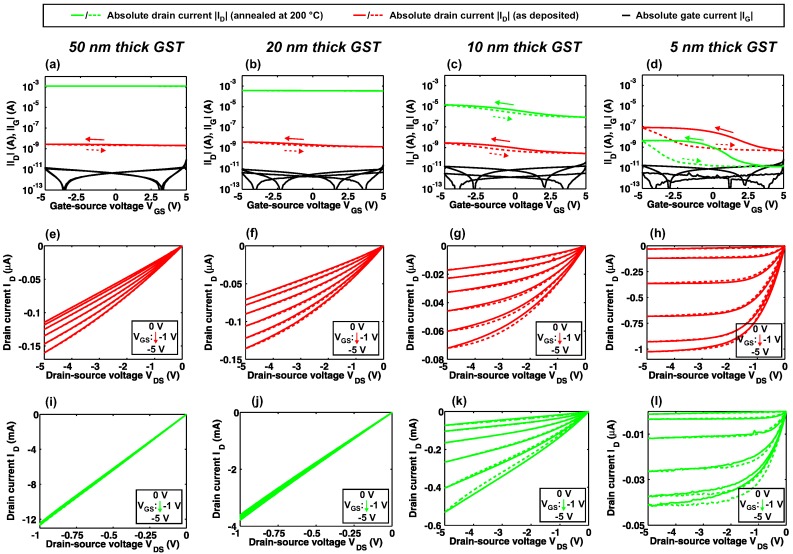
Electrical characteristics of GST thin-film transistors (TFTs) with channel lengths of 10 μm and a channel widths of 100 μm. Transfer characteristics at a drain-source voltage V${}_{\mathrm{DS}}$ = −100 mV for (**a**) 50 nm, (**b**) 20 nm, (**c**) 10 nm and (**d**) 5-nm-thick GST. Output characteristics of amorphous (as deposited) GST TFTs for (**e**) 50 nm, (**f**) 20 nm, (**g**) 10 nm and (**h**) 5-nm-thick GST. Output characteristics of annealed GST TFTs for (**i**) 50 nm, (**j**) 20 nm, (**k**) 10 nm and (**l**) 5-nm-thick GST.

In Figure 3, the electrical characteristics of 5–50-nm-thick GST TFTs are compared. All TFTs display the expected p-type behavior with increasing $|I_{D}|$ for negative V${}_{\mathrm{GS}}$. The transfer characteristics of 50 nm and 20-nm-thick GST TFTs show the desired $|I_{D}|$ increase upon annealing, however, nearly all I${}_{D}$ modulation capabilities are lost (see Figure 3a,b). With decreasing GST thickness, the I${}_{D}$ ON/OFF modulation is improved (Figure 3a–d). Furthermore, the significantly increased $|I_{D}|$ at V${}_{\mathrm{GS}}$ = −5 V for 5-nm-thick amorphous GST TFTs (≈20x compared to thicker amorphous layers) leads to the largest ON/OFF ratio of ≈300 at this GST thickness. The annealing of these ultra-thin GST films results in an $|I_{D}|$ decrease, which can be attributed to a degradation of the channel and contact area as discussed later. The output characteristics of amorphous GST TFTs (Figure 3e–h) show an increasingly saturating behavior for reduced GST thicknesses. The strongly saturated characteristics for 5-nm-thick amorphous GST TFTs indicate that the channel pinch-off is successfully obtained. After annealing, the thick GST films ≥20 nm are highly conductive and the TFTs show ohmic behavior with large $|I_{D}|$ (Figure 3i,j). We found that large V${}_{\mathrm{DS}}$ lead to a breakdown of the these devices and thus the maximum V${}_{\mathrm{DS}}$ was set to 1 V. Nevertheless, the TFTs with GST thicknesses ≤10 nm after annealing can sustain larger V${}_{\mathrm{DS}}$ (Figure 3k,l). A fully saturating I${}_{D}$ was only obtained for 5-nm-thick annealed GST semiconductor layers, which is similar to the findings in the as deposited state.

In Table 1, the threshold voltages (V${}_{\mathrm{Th}}$) and subthreshold swings (SS) of the devices are presented. Both measures monotonously rise with increasing GST thickness. It has to be noted that due to the low ON/OFF ratio, which is further evaluated below, the values for TFTs with 50-nm-thick amorphous (as deposited) GST channels are impractical. For the same reason, no V${}_{\mathrm{Th}}$ or SS could be extracted for TFTs with GST thicknesses ≥20 nm after annealing.

**Figure 4:**
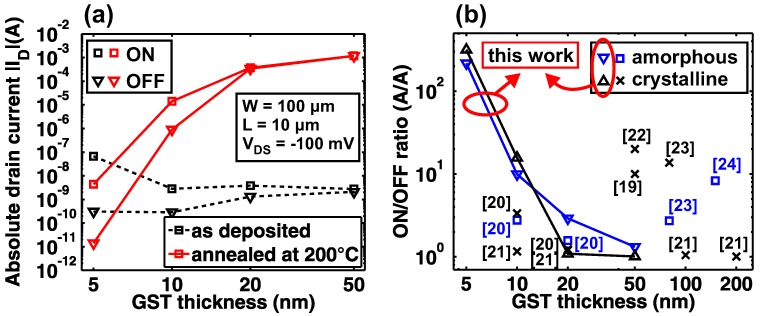
Comparison of the ON-currents and OFF-currents for different GST thicknesses. (**a**) ON- and OFF-currents. (**b**) ON/OFF ratio with a comparison to literature using [19,20,21,22,23,24].

In Figure 4a, we compare the TFT ON-currents ($|I_{D,\mathrm{ON}}|$ at V${}_{\mathrm{GS}}$ = −5 V) and OFF-currents ($|I_{D,\mathrm{OFF}}|$ at V${}_{\mathrm{GS}}$ = +5 V) for different GST thicknesses. For amorphous (as deposited) GST, the $|I_{D,\mathrm{OFF}}|$ can be reduced when scaling the thickness to ≤10 nm and the $|I_{D,\mathrm{ON}}|$ exhibits an increase between 10 nm and 5 nm of GST. As shown in Table 2, the effective field-effect mobilities $\mu_{\mathrm{FE},\mathrm{eff}}$ agree with these trends and have values in the expected range [27,34,35]. The annealing provides the desired $|I_{D}|$ increase only down to 10 nm of GST below which the post-annealing $|I_{D}|$ deteriorates. The $\mu_{\mathrm{FE},\mathrm{eff}}$ values for 20 and 50 nm of annealed GST cannot be extracted due to the low ON/OFF ratio, and for 10-nm-thick GST TFTs we obtain $\mu_{\mathrm{FE},\mathrm{eff}}$ = 6.7 cm${}^{2}$/Vs. Further, we performed Hall measurements on the crystallized GST films (see Table 2). The amorphous films were too resistive for the Hall measurements. We obtained Hall mobilities around ≈3 cm${}^{2}$/Vs and carrier concentrations in the order of 10${}^{20}$ cm${}^{-3}$, which is in agreement with prior findings for fcc-GST [34,35]. Figure 4b displays the ON/OFF ratio for different GST thicknesses with a literature comparison. Other works have so far not investigated GST TFTs with active layer thicknesses below 10 nm. In contrast to prior work, we find a clear trend indicating an increasing ON/OFF ratio with reduced GST film thickness. Our maximum ON/OFF ratio of 316 for 5-nm-thick annealed GST results in a ≈15-fold improvement compared to prior reports. The strong thickness dependence of the ON/OFF ratio and the significant improvement for ultra-thin GST layers can be attributed to the electronic energetics of GST in both the amorphous and crystalline phases. For both situations, the gate electric field has a limited penetration depth into the GST layer. For the amorphous phase, GST exhibits a large number of acceptor-like and donor-like traps, which compensate each other leading to strong Fermi-level pinning in the middle of the band gap [36,37,38]. Additionally, there are Te lone-pairs, which could also be responsible for the observed hysteresis in the transfer characteristics (Figure 3a–d) [36,37]. In the crystalline phase, mainly acceptor-type vacancy defects are present [36,37], which results in strong p-type doping and a Fermi-level shift into the valence band forming a degenerate semiconductor [35,38]. In this case, the depletion width for the gate electric field is strongly limited [39] and a fully-depleted TFT can only be achieved for ultra-thin semiconductor layers. Thus, the OFF-current of GST TFTs is a strong function of GST thickness and can be reduced with thinner GST layers. In the following, the dependence of the ON-current on the GST thickness is discussed.

**Figure 5:**
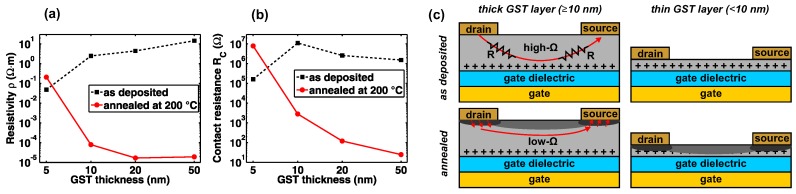
Influence of GST thickness and annealing on resistivity and contact resistance. (**a**) Resistivity ρ. (**b**) Contact resistance R${}_{C}$. (**c**) Sketch visualizing the proposed mechanisms.

For that, we analyze TFTs with different channel lengths between 7 μm and 100 μm to extract the resistivity ρ (Figure 5a) and contact resistance R${}_{C}$ (Figure 5b). For the amorphous case, we find that the ρ and R${}_{C}$ are strongly reduced for 5-nm-thick GST compared to thicker layers. This can be attributed to a parasitic resistance of the highly resistive GST film [40,41], due to the limited penetration depth of the gate electric field forming a very thin conductive accumulation region (see Figure 5c, top-left) [42,43]. Thus, the ON-current in TFTs with amorphous GST channels can be improved by thickness downscaling due to a reduction of the parasitic series resistances. In contrast, the annealed GST thin-film exhibits a significant decrease in ρ and R${}_{C}$ for GST thicknesses ≥10 nm, which is related to the large conductivity of the crystalline phase. We attribute the unsuccessful reduction of ρ and R${}_{C}$ upon annealing for 5-nm-thick GST to a degradation of the contact and surface area, which could be caused by an oxidation during annealing and migration effects in the source/drain contact area. We believe that these effects should certainly have an increased impact for thinner GST channels as indicated in dark gray in Figure 5c (bottom). To confirm that the device oxidation is responsible for the degradation of ρ, future work should address different annealing parameters and environment such as higher vacuum level or nitrogen atmosphere.

**Figure 6:**
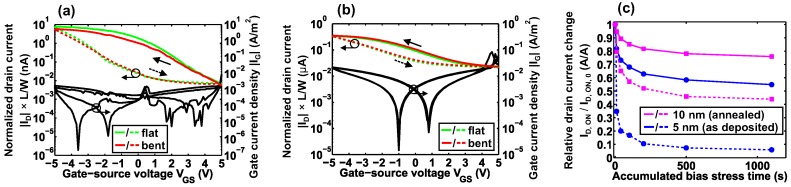
Bending and bias stress measurements. (**a**) Transfer characteristic of a TFT with a 5-nm-thick amorphous (as deposited) GST channel under bending (radius R = 6 mm). (**b**) Transfer characteristic of a TFT with a 10-nm-thick crystallized (annealed) GST channel under bending (radius R = 6 mm). (**c**) ON-current $I_{D,\mathrm{ON}}$ change upon bias stress. The dashed lines represent $I_{D,\mathrm{ON}}$ directly after bias stress and the solid lines correspond to $I_{D,\mathrm{ON}}$ after a sweep to positive V${}_{\mathrm{GS}}$.

From the above described measurements and analysis, we conclude that among the amorphous (as deposited) and crystallized (annealed) devices the TFTs with 5-nm-thick GST and 10-nm-thick GST show the highest performance, respectively. Thus, we performed bending tests with these devices by wrapping the polyimide substrates around a metallic rod with a radius R = 6 mm. Figure 6a,b show the results of the bending tests for TFTs with 5-nm-thick amorphous GST channels and 10-nm-thick crystallized GST channels, respectively. Both devices remain fully operational during the bending experiments. The former (a) shows a reduction of $|I_{D,\mathrm{ON}}|$ and $\mu_{\mathrm{FE},\mathrm{eff}}$ [44] while the latter (b) remains almost unaltered with a small $|I_{D,\mathrm{ON}}|$ increase. From prior findings, we know that, typically, devices on 50 μm thick polyimide sustain bending to R = 5–6 mm without any significant alterations in their characteristics [45,46,47]. However, the here observed $|I_{D,\mathrm{ON}}|$ reduction by ≈25% for TFTs with 5-nm-thick amorphous GST channels indicates that not the bending, but the preceding electrical characterization may have caused this change. Thus, we investigated the changes in $|I_{D,\mathrm{ON}}|$ upon bias stress measurements (see Figure 6c). After a cumulative bias stress time of 1100 s, the TFT with a 5-nm-thick amorphous GST channel has an $|I_{D,\mathrm{ON}}|$, which is reduced to less than 10% of its initial value, which is then partially recovered to about 60% after a sweep to positive V${}_{\mathrm{GS}}$. In contrast, the changes for the TFT with a 10-nm-thick crystallized GST channel are significantly smaller. A reason for the strong bias stress dependence of the TFTs with amorphous channels could be intrinsic defects such as Te lone-pairs [36,37], which we also attributed to the observed hysteresis in the transfer characteristics. These defects are not expected to be present in the crystalline phase of GST. Consequently, we see less bias stress dependence in the crystallized films. However, due to the mixed crystalline phase of GST (see Figure 2) after annealing, there could still be Te lone-pairs present e.g., at grain boundaries. An additional effect, which needs to be taken into account, is the known resistance drift over time in amorphous GST, which could also partially cause the $|I_{D,\mathrm{ON}}|$ reduction [48,49].

## 4. Conclusions and Outlook

We have reported a significant performance improvement for GST TFTs by lowering the semiconductor thickness down to 5 nm. The ON/OFF-ratio was improved by a factor of ≈15 and saturating output characteristics were obtained. Although a $\mu_{\mathrm{FE},\mathrm{eff}}$ of 6.7 cm${}^{2}$/Vs for 10-nm-thick crystalline GST TFTs was observed, the abovementioned performance improvements could only be achieved at a GST thickness of 5 nm, where the maximum $\mu_{\mathrm{FE},\mathrm{eff}}$ was found to be 0.04 cm${}^{2}$/Vs. This work shows that GST is a promising p-semiconductor for flexible electronics. Thus, we recently demonstrated the integration of such TFTs with n-type InGaZnO${}_{4}$ to form flexible CMOS circuits [44]. Therein, we showed a bending stability of these circuits to a tensile radius of 6 mm.

However, several aspects still need to be addressed in the future. The crystallization process for 5-nm-thick GST films needs improvement, which could be achieved by changing the crystallization temperature, atmosphere and time, and by applying appropriate passivation layers to GST prior to the crystallization. In addition, different source/drain contact materials could be studied to investigate their impact on R${}_{C}$ before and after annealing. Furthermore, other material compositions could be investigated. The electronic properties of crystalline GST could be altered by increasing the Ge content, which could reduce the number of Ge vacancies responsible for the p-type doping [50]. The resulting reduction of free carriers could then increase the minimum GST thickness required for the improved TFT performance, and thus the crystallization process could be simplified. Furthermore, a significant increase of the Sb content has resulted in reduced resistivities in the amorphous phase of GST [51], which could be a path for improvements of TFTs with amorphous GST channels.

## Figures and Tables

**Table 1:** Threshold voltages (V${}_{\mathrm{Th}}$) and subthreshold swings (SS) of the GST TFTs extracted at a drain-source voltage V${}_{\mathrm{DS}}$ = −100 mV.

GST Thickness (nm)	V${}_{\mathrm{Th}}$ (V) as Deposited	V${}_{\mathrm{Th}}$ (V) Annealed at 200 °C	SS (V/Decade) as Deposited	SS (V/Decade) Annealed at 200 °C
5	0.86	0.6	1.3	1.0
10	1.8	1.72	5.4	5.1
20	4.56	-	13	-
50	20.55	-	22	-

**Table 2:** Effective field-effect mobility $\mu_{\mathrm{FE},\mathrm{eff}}$ (cm${}^{2}$/Vs) for TFTs with different GST thicknesses extracted in the linear regime and Hall mobilities $\mu_{\mathrm{Hall}}$ (cm${}^{2}$/Vs) and carrier concentrations *n* (cm${}^{-3}$) measured in GST thin-films.

GST Thickness (nm)	$\mu_{\mathrm{FE},\mathrm{eff}}$ as Deposited	$\mu_{\mathrm{FE},\mathrm{eff}}$ Annealed at 200 °C	$\mu_{\mathrm{Hall}}$ Annealed at 200 °C	*n* Annealed at 200 °C
5	0.041	0.0032	-	-
10	0.0014	6.72	2.79	+9.6$\times 10^{19}$
20	0.0013	-	3.7	+4.1$\times 10^{20}$
50	0.0004	-	2.92	+7.2$\times 10^{20}$
